# An experimental pig model with outer retinal degeneration induced by temporary intravitreal loading of *N*-methyl-*N*-nitrosourea during vitrectomy

**DOI:** 10.1038/s41598-020-79437-1

**Published:** 2021-01-08

**Authors:** Kwang-Eon Choi, Vu Thi Que Anh, Jee Taek Kim, Cheolmin Yun, Seongkwang Cha, Jungryul Ahn, Yong Sook Goo, Seong-Woo Kim

**Affiliations:** 1 Department of Ophthalmology, Korea University College of Medicine, Seoul, South Korea; 2 Department of Ophthalmology, Hanoi Medical University, Hanoi, Vietnam; 3 Department of Ophthalmology, Chung-Ang University College of Medicine, Seoul, South Korea; 4 Department of Physiology, Chungbuk National University College of Medicine, Cheongju, South Korea

**Keywords:** Retina, Macular degeneration, Retinal diseases

## Abstract

We aimed to develop an outer retinal degeneration pig model induced by temporary intravitreal loading of *N*-methyl-*N*-nitrosourea (MNU) during vitrectomy. In a preliminary experiment involving 5 mini-pig cases to determine the appropriate concentration of MNU, the vitreous cavity of each eye was filled with 4, 8, 10, 12, or 16 mg/mL MNU for 10 min, which was then replaced with a balanced salt solution. Multimodal examinations including spectral-domain optical coherence tomography (OCT) images and full-field electroretinography (ffERG) were obtained at baseline and week 2, week 6, and week 12. The retinal degeneration was classified according to the amplitudes of a dark adaptive (DA) 10.0 a-wave amplitude. The degree of moderate retinal degeneration was defined as DA 10.0 a-wave amplitude ≥ 10% and < 60% of baseline amplitude. The degree of severe degeneration was defined as DA 10.0 a-wave amplitude < 10% of baseline amplitude, noise, or flat signal. Hematoxylin and eosin staining and immunohistochemistry were performed at week 12. The main experiments were conducted first with 10 cases of 5 mg/mL and later with 13 cases of 10 mg/mL. In the preliminary experiment, degree of outer retinal degeneration increased with MNU concentration. Use of 4, 8, 10, 12, and 16 mg/mL MNU showed no, moderate, severe, severe, and atrophic changes, respectively. In the main experiments, there were 9 cases of moderate retinal degeneration and 1 case of severe degeneration in 5 mg/mL MNU group. Two cases of moderate degeneration and 11 of severe degeneration were recorded in 10 mg/mL group. Mean thickness of total retina, inner nuclear layer, and outer nuclear layer decreased at week 2 in both groups. The mean amplitudes on ffERG decreased at week 2. The ffERG and OCT findings did not change from week 2 to week 6 or week 12. The results of staining supported those of ffERG and OCT. Temporal MNU loading in a vitrectomized pig-eye model induced customized outer retinal degeneration with changing the concentration of MNU.

## Introduction

Retinal degeneration and dry age-related macular degeneration, such as retinitis pigmentosa and geographic atrophy, are the important causes of blindness^1,2^. To treat patients, retinal prosthetics such as Argus II (SECOND SIGHT, Sylmar, CA, USA) and alpha AMS (Retina Implant AG, Reutlingen, Germany) have been implanted for investigational or commercial purposes in many countries^3–5^. While neither Argus II nor alpha AMS are being manufactured anymore, other retinal implants have been developed^6,7^. To accelerate development of retinal implants, the efficacy and safety of the retinal implants must be tested at the preclinical stage. Animal models physiologically similar to human outer retinal degeneration are important.

Knock-out animal models offer relevant similarities to human eye^8–13^. However, these models are expensive and can require prolonged periods to manifest disease. Furthermore, there is limited ability of gene knock-out models to control the onset of disease and determine its severity at a certain age^10,11^.

Pharmaceutical animal models can substitute for genetic models with respect to price and relative ease of mass production. These models have been induced by systemic application of pharmaceuticals such as *N*-methyl-*N*-nitrosourea (MNU), iodoacetic acid, and sodium iodate (SI)^14–17^. However, for a large animals, few models of retinal degenerations have been successfully induced by systemic injection of drugs^15,18,19^. Toxic pharmaceuticals can result in systemic toxicity and deterioration of animal general health^20,21^. To avoid these problems, intravitreal injection of small doses of pharmaceuticals has been tested, and positive results have been reported^22^. In previous studies, we successfully induced outer retinal degeneration by intravitreal injection of SI or MNU after vitrectomy in rabbit and dog^23,24^. While globally diffuse homogeneous outer retinal degeneration can be acquired in a vitrectomized eye with SI, intravitreal MNU injection induced only localized homogeneous retinal degeneration, even in a vitrectomized eye. One difficulty was that dose of intravitreally-injected drug must be adjusted and defined according to eyeball size or lens status of each different animal. To solve this problem, the drug administration protocol need to be changed from a volume-based method to one based on concentration. The purpose of the present study was to induce globally homogeneous outer retinal degeneration with a temporary MNU tamponade in the vitreous cavity and to correlate intravitreal MNU concentration with degree of retinal degeneration in a large animal (mini-pig).

## Results

### Animals

The mean age of mini-pigs was 11.13 ± 2.31 months, and the mean body weight was 29.86 ± 2.94 kg. The mean axial length was 20.32 ± 0.81 mm. During follow-up periods, there was no significant weight loss and decrease of activity in all mini-pigs.

### The first preliminary experiment of each dose of MNU

#### Full-field electroretinography and multifocal electroretinography

**Figure 1:**
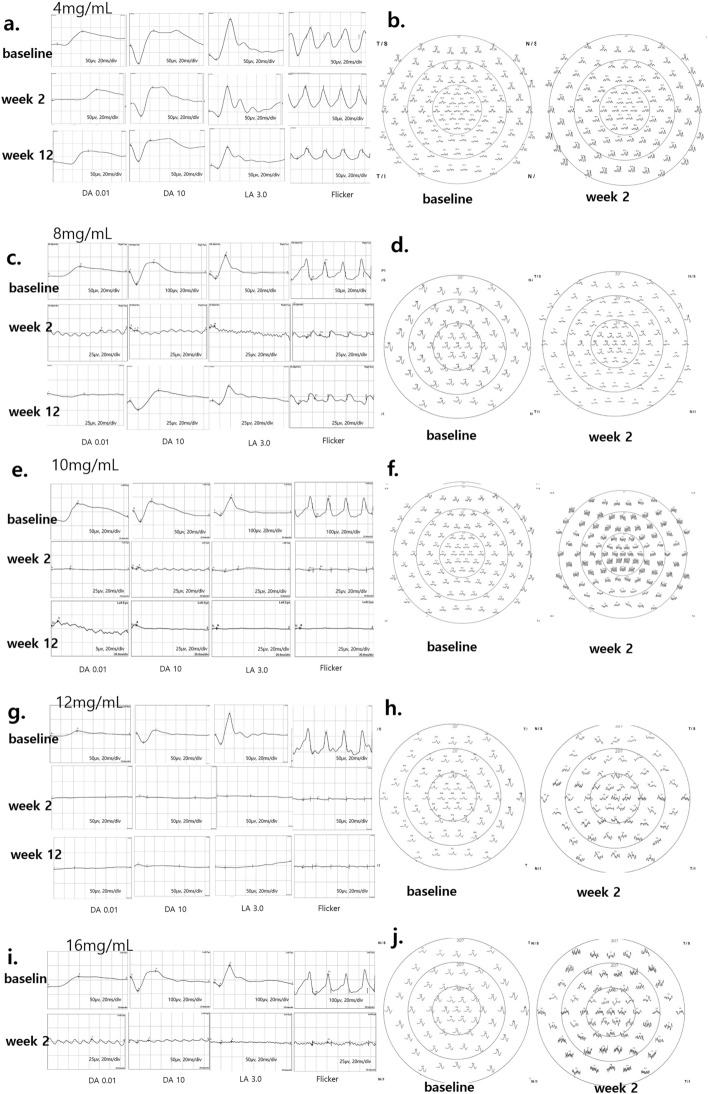
ffERG and mfERG findings of the retina in one case of each 4 mg/mL, 8 mg/mL, 10 mg/mL, 12 mg/mL, and 16 mg/mL MNU. (**a**) The DA 10.0 a-wave amplitude at baseline and week 2 was 25.4 µV and 26.1 µV, respectively, in the 4 mg/mL MNU case. (**b**) mfERG in the 4 mg/mL MNU showed no signal flattening or noise signals at week 2. (**c**) The DA 10.0 a-wave amplitude at baseline and week 2 was 89.3 µV and 10.1 µV, respectively, in the 8 mg/mL MNU case. The amplitudes in scotopic and photopic ffERG decreased at week 2, but no signal was flat or appeared as noise, especially in photopic ffERG. (**d**) mfERG in the 8 mg/mL MNU case showed focal noise signal and signal flattening at week 2. (**e**) The DA 10.0 a-wave amplitude at baseline and week 2 was 38.6 µV and 6.21 µV, respectively, in a 10 mg/mL MNU case. All signals of ffERG showed nearly flat down signals or noise signals at week 2. (**f**) The mfERG in a 10 mg/mL MNU case showed only noise signals at week 2. (**g**) The DA 10.0 a-wave amplitude at baseline and week 2 was 61.3 µV and 5.67 µV, respectively, in a 12 mg/mL MNU case. All signals of ffERG showed nearly flat down signals or noise signals at week 2. (**h**) The mfERG in a 12 mg/mL MNU case showed only noise signals at week 2. (**i**,**j**) Both ffERGs and mfERGs in a 16 mg/mL MNU case showed noise signals at week 2. *ffERG *full field electroretinogram, *MNU N*-methyl-*N*-nitrosourea, *DA *dark adaptive, *mfERG *multifocal electroretinogram.

The 4 mg/mL MNU case showed no degeneration according to ffERG results. No decrease of amplitude and no delay of implicit time was evident at week 2 in the scotopic and photopic ffERG results. No flat or noise signals were seen in the mfERG. The 8 mg/mL MNU case showed moderate degeneration under ffERG. The amplitude decreased after 2 weeks in the scotopic and photopic ffERG results. However, only noise was observed at week 2 in mfERG analysis. Both the 10 mg/mL and 12 mg/mL MNU cases showed severe degeneration under ffERG. All ffERG results showed an almost flat down signal or noise signal. Only noise was observed in mfERG. Finally, the 16 mg/mL MNU case showed noise signals for both ffERG and mfERG at 12 weeks after MNU exposure (Fig. 1, Supplementary Fig. 1).

#### Fundus infrared reflectance and spectral-domain optical coherence tomography

**Figure 2:**
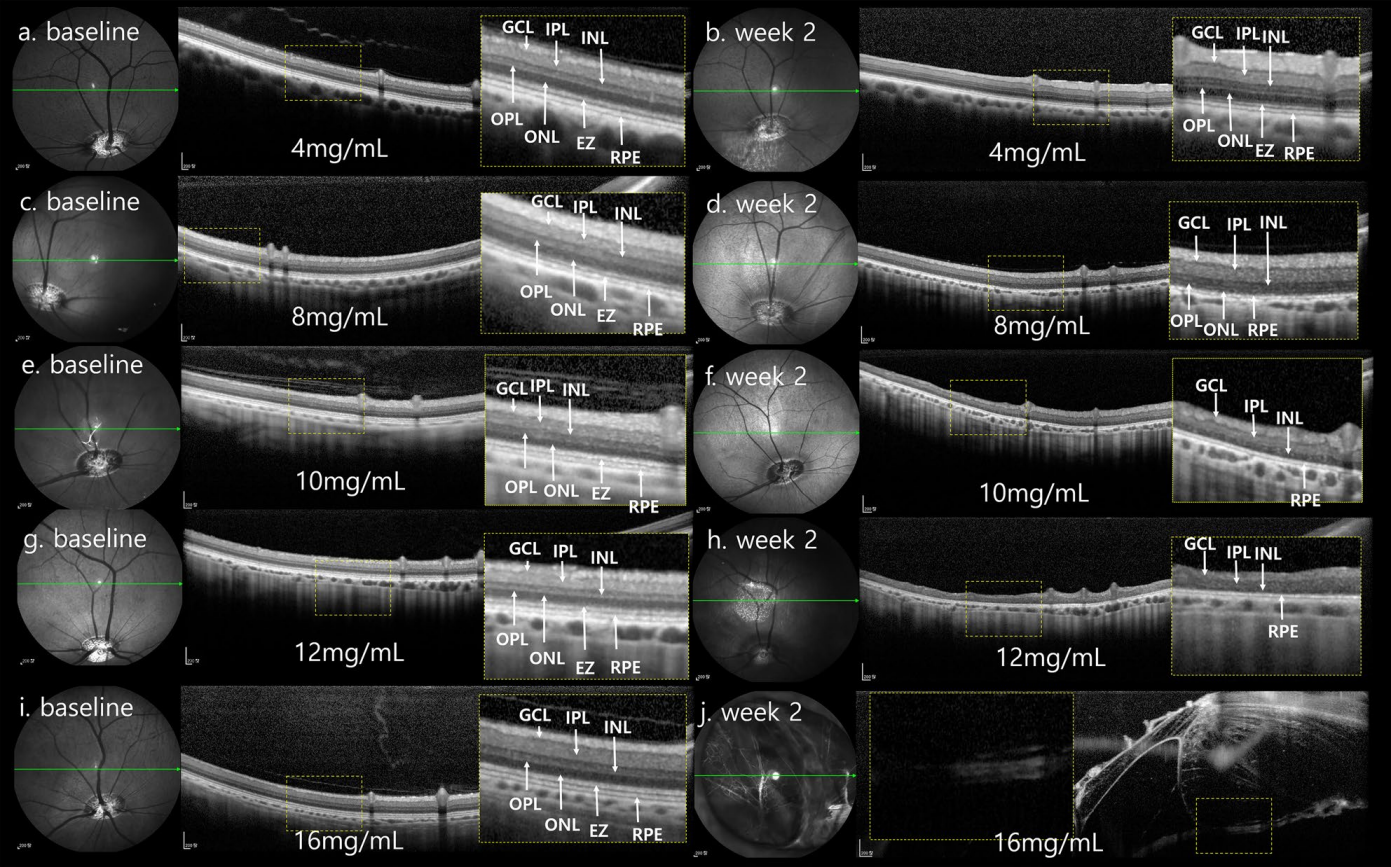
SD-OCT findings of the retina in one case of each 4 mg/mL, 8 mg/mL, 10 mg/mL, 12 mg/mL, and 16 mg/mL MNU at baseline and week 2. (**a**–**j**) Each magnified SD-OCT image of the case is on the right sided and shown with a dashed-line box. (**a**,**b**) OCT image of the 4 mg/mL MNU case showed intact GCL, INL, ONL, photoreceptor layer, and RPE at baseline and week 2. (**c**,**d**) OCT image of the 8 mg/mL MNU case at week 2 showed an intact ganglion cell layer and retinal pigment epithelium. The boundary of the INL and ONL became indistinct compared with the SD-OCT image at baseline (**c**). The EZ representing the photoreceptor layer became obscure. OCT images of the 8 mg/mL MNU case at weeks 6 and 12 weeks showed no change compared with that at week 2 (Supplementary Fig. 2). (**e**,**f**) On the OCT, unlike baseline, the EZ and ONL were indistinguishable at week 2 in the 10 mg/mL MNU case. However, there was no significant decrease in INL thickness. (**g**,**h**) On the OCT, unlike baseline, the EZ and ONL were indistinguishable at week 2 in the 12 mg/mL MNU case. In addition, there was a decrease in INL thickness. The total retinal thickness of the 12 mg/mL MNU case at week 6 was thinner than at week 2 (Supplementary Fig. 2). (**i**,**j**) After week 2, the retina melted and induced retinal detachment in SD-OCT images of the 16 mg/mL MNU case. *SD-OCT *spectral domain optical coherence tomography, *MNU N*-methyl-*N*-nitrosourea, *GCL *ganglion cell layer, *IPL *inner plexiform layer, *INL *inner nuclear layer, *OPL *outer plexiform layer, *ONL *outer nuclear layer, *EZ *ellipsoid zone, *RPE *retinal pigment epithelium.

The 4 mg/mL MNU case showed that the 3 nuclear layers of ganglion cell layer (GCL), ONL, and INL were well maintained at week 2. The EZ and RPE layers were also intact in the 4 mg/mL MNU case. In the 8 mg/mL MNU case, the boundary of INL and ONL became indistinct at week 2, and the EZ became obscure. Both the 10 mg/mL and 12 mg/mL MNU cases showed indistinguishable EZ and ONL at week 2. However, the 12 mg/mL MNU case showed a decrease of INL thickness more than the 10 mg/mL MNU case at week 2 compared with baseline. These trends remained stable until weeks 6 and 12 in the 4 mg/mL, 8 mg/mL, 10 mg/mL and 12 mg/mL MNU cases. In the 16 mg/mL MNU case, the layers became atrophic and the focal retina melted, causing focal retinal detachment at week 2 (Fig. 2, Supplementary Fig. 2).

#### Hematoxylin and eosin and immunohistochemistry

**Figure 3:**
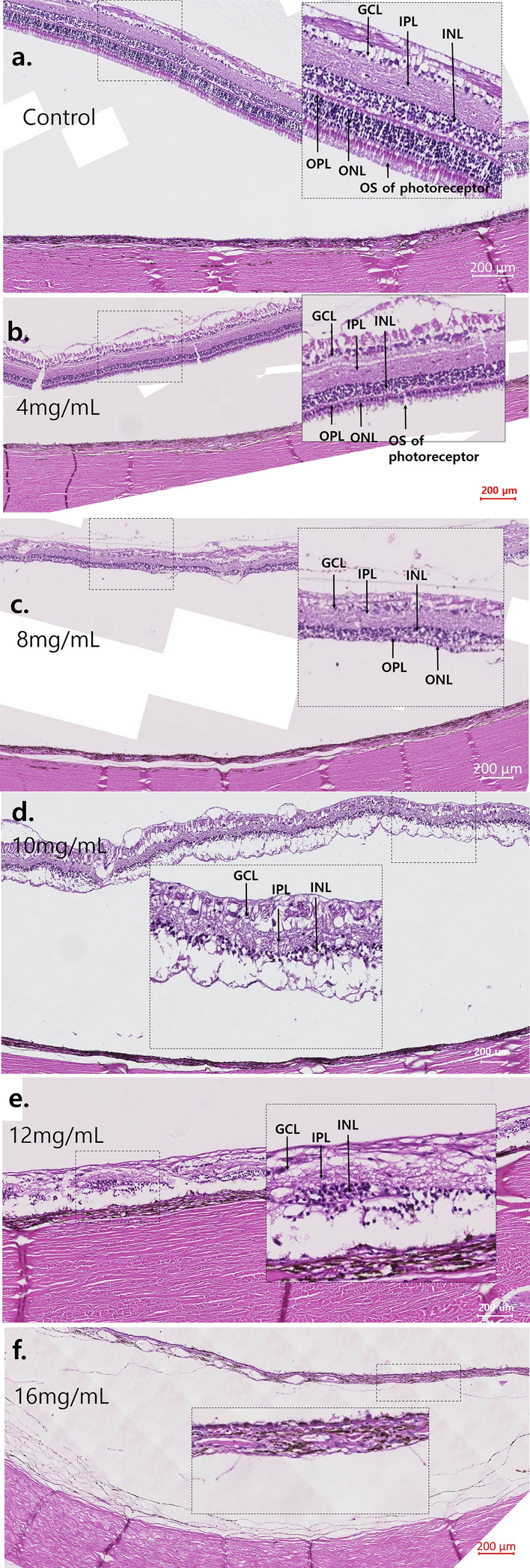
H&E staining image of the retina in cases of 4 mg/mL, 8 mg/mL, 10 mg/mL, 12 mg/mL, and 16 mg/mL MNU. (**a**–**f**) Each magnified H&E staining image of the case is on the right-side shown with a dashed-line box. (**a**) H&E staining of the control case. Each count number of ONL, INL, and GCL in dotted enlarged area was 586, 298, and 56, respectively. (**b**) In H&E staining of the 4 mg/mL MNU case, all layers including the INL, GCL, and NFL were nearly intact. However, the thickness of OPL and ONL decreased. ONL (4 nuclear layers) of the 4 mg/mL MNU case is thinner than ONL (7–8 nuclear layers) of control (**a**). Each count number of ONL, INL, and GCL in dotted enlarged area was 247, 285, and 50, respectively. (**c**) In H&E staining of the 8 mg/mL MNU case, the outermost photoreceptors remained to some degree but at severely reduced density. The density of ONL and INL also was reduced, but the GCL was relatively intact. Each count number of ONL, INL, and GCL in dotted enlarged area was 43, 216, and 40, respectively. (**d**) In H&E staining of the 10 mg/mL MNU case, ONL cells were rarely observed among INL or combined INL with ONL cells. Each count number of ONL, INL, and GCL in dotted enlarged area was 0, 87, and 16, respectively. (**e**) In H&E staining of the 12 mg/mL MNU case, ONL cells were rarely observed among INL or combined INL with ONL cells. Each count number of ONL, INL, and GCL in dotted enlarged area was 0, 74, and 15, respectively. (**f**) In H&E staining of the 16 mg/mL MNU case, proliferative vitreoretinopathy and retinal detachment were observed, and the entire layer was extremely thin. *MNU N*-methyl-*N*-nitrosourea, *GCL *ganglion cell layer, *IPL *inner plexiform layer, *INL *inner nuclear layer, *OPL *outer plexiform layer, *ONL *outer nuclear layer, *OS *outer segment.

**Figure 4:**
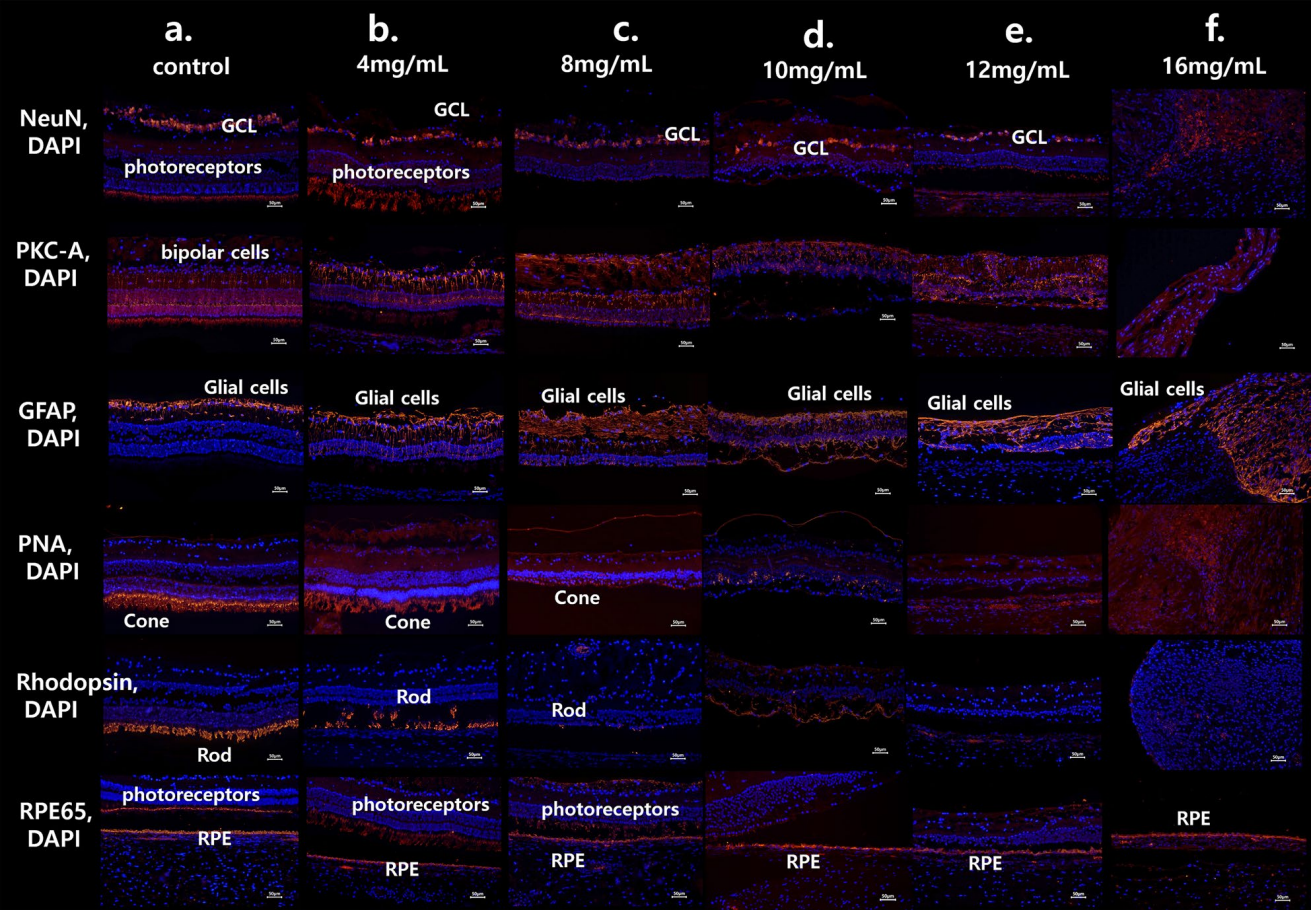
The immunohistochemistry findings of the retina in cases of 4 mg/mL, 8 mg/mL, 10 mg/mL, 12 mg/mL, and 16 mg/mL MNU. (**a**) The normal immunohistochemistry of a control case. Rhodopsin staining and PNA staining showed well-stained rod and cone cells (each percentage of the stained layer = 12.86% and 13.51%). (**b**) Immunohistochemistry of the 4 mg/mL MNU case. NeuN stain showed intact ganglion cells and co-staining of photoreceptors. PKC-α staining showed intact bipolar cells, and GFAP staining showed nearly normal muller cells. Rhodopsin staining showed intact rod cells (the percentage of the stained layer = 10.03%), and PNA staining showed intact cone cells (the percentage of the stained layer = 12.44%). RPE65 staining showed intact RPE and co-staining of photoreceptors. **c**. Immunohistochemistry of the 8 mg/mL MNU case. NeuN staining showed intact GCL. PKC-α staining showed nearly intact bipolar cells but co-staining of few photoreceptors. GFAP staining showed mildly increased stained cells. Rhodopsin staining and PNA staining showed few stains of rod and cone cells, respectively (each percentage of the stained layer = 0.23% and 3.22%). RPE65 showed nearly intact RPE cells with co-staining of a few photoreceptors. (**d**) Immunohistochemistry of the 10 mg/mL MNU case. NeuN and PKC-α stainings showed ganglion cells and bipolar cells, respectively. There was no co-staining of photoreceptors with NeuN staining. GFAP stain showed increased staining. Rhodopsin and PNA staining showed rarely stained rod and cone cells (each percentage of the stained layer = 0.43% and 2.11%).with co-staining of RPE cells. RPE 65 showed intact RPE cells. (**e**) Immunohistochemistry of the 12 mg/mL MNU case. NeuN and PKC-α stainings showed ganglion cells and bipolar cells, respectively. There was no co-staining of photoreceptors with NeuN staining. GFAP stain showed increased staining. Rhodopsin and PNA staining showed no stained rod and cone cells (each percentage of the stained layer = 0% and 0%) with co-staining of RPE cells. RPE 65 showed intact RPE cells. (**f**) Immunohistochemistry of the 16 mg/mL MNU case. RPE staining showed nearly intact RPE cells, and GFAP staining showed a marked increase in stained lesions. *MNU N*-methyl-*N*-nitrosourea, *GCL *ganglion cell layer, *IPL *inner plexiform layer. Each percentage of the stained layers was evaluated by the stained area/all layer area using ImageJ software (1.53a version, National Institutes of Health, Bethesda, MD, USA).

The 4 mg/mL MNU case showed that all INL, GCL, and nerve fiber layer (NFL) were intact. Photoreceptors were nearly intact in H&E staining (Fig. 3). For Immunohistochemistry, Rhodopsin staining, and PNA staining showed intact cone and rod cells. RPE65 staining showed intact RPE and co-staining of photoreceptors. NeuN staining showed intact ganglion cells and co-staining of photoreceptors. PKC-α staining showed intact bipolar cells, and GFAP staining showed nearly normal Müller cells (Fig. 4).

The density of photoreceptors decreased in H&E staining of the 8 mg/mL MNU sample (Fig. 3). The densities of ONL and INL were also reduced, while GCL was relatively intact. Rhodopsin staining and PNA staining showed few remnant rods and cones, respectively. RPE65 and PKC-α staining showed nearly intact RPE cells and bipolar cells. NeuN staining showed intact GCL but co-staining of few photoreceptors. GFAP staining showed mildly increased staining (Fig. 4).

The 10 mg/mL MNU case revealed few ONL cells among mainly INL cells in H&E staining (Fig. 3). Rhodopsin and PNA staining showed rare rod or cone cells with co-staining of RPE cells. RPE 65, PKC-α, and NeuN staining showed intact RPE cells, bipolar cells, and ganglion cells, respectively. No co-staining of photoreceptors was evident in NeuN staining. GFAP staining showed increased staining (Fig. 4).

The 12 mg/mL MNU case revealed no ONL cells among mainly INL cells in H&E staining (Fig. 3). Rhodopsin and PNA staining showed no rod or cone cells with co-staining of RPE cells. RPE 65, PKC-α, and NeuN staining showed intact RPE cells, bipolar cells, and ganglion cells, respectively. No co-staining of photoreceptors was evident in NeuN staining. GFAP staining showed increased staining (Fig. 4).

The 16 mg/mL MNU case revealed proliferative vitreoretinopathy and retinal detachment, and all layers were extremely thin in H&E staining (Fig. 3). In immunohistochemistry, RPE staining showed nearly intact RPE cells (Fig. 4).

### Efficacy experiment results of the 5 mg/mL MNU group (n = 10)

#### Full-field electroretinography and multifocal electroretinography

**Figure 5:**
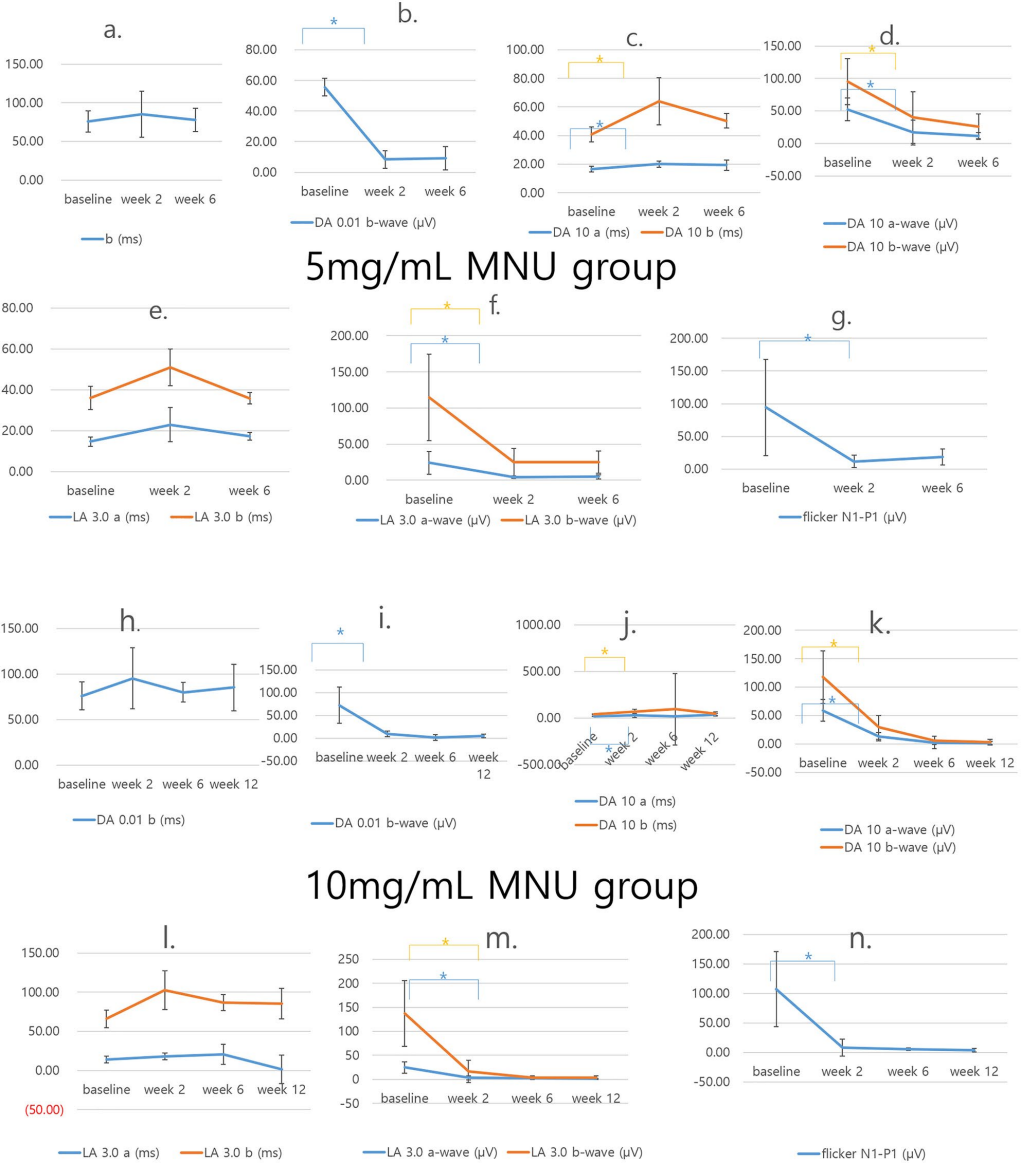
The ffERG results of the 5 mg/mL MNU group (n = 10) and the 10 mg/mL MNU group (n = 13). (**a**–**g**) The ffERG of the 5 mg/mL MNU group. (**a**,**b**) The 5 mg/mL MNU group showed delayed implicit time of b-waves in dark adaptive (DA) 0.01 ERG at weeks 2 and and 6, but the change was not statistically significant. The 5 mg/mL MNU group showed significant decrease of b-wave in dark adaptive (DA) 0.01 ERG from baseline to week 2 (p = 0.028). (**c**,**d**) In DA 10.0 ERG, the 5 mg/mL MNU group showed delay of a- and b-waves at week 2 (p = 0.028, 0.018, respectively) and appeared to recover somewhat at week 6, but the amplitudes of both a- and b-waves decreased at week 2 (p = 0.005, 0.005, respectively) and remained unchanged from week 2 to week 6. (**e**,**f**) In light adaptive (LA) 3.0 ERG, the 5 mg/mL MNU group showed decrease in a- and b-wave amplitudes at week 2 (p = 0.018, 0.018, respectively) and remained unchanged at week 6, but the implicit time of both a- and b-waves did not change during follow-up periods. (**g**) The flicker amplitude in the 5 mg/mL MNU group showed a decrease in amplitude at week 2 (p = 0.028), but further decrease was not statistically significant. (**h–n**) The ffERG results of the 10 mg/mL MNU group. (**h**,**i**) The b implicit time in the DA 0.01 ERG did not show a significant change in the 10 mg/mL MNU group, but the amplitude was nearly flattened at week 2 (p = 0.008) and showed no recovery or variation after. (**j**,**k**) The b implicit time in the DA 10 ERG show a significant change in the 10 mg/mL MNU group (p = 0.008, 0.008, respectively), and the amplitude nearly flattened at week 2 (p = 0.008, 0.008, respectively) and showed no recovery or variation after that point. (**l**,**m**) In LA 3.0 ERG, the 10 mg/mL MNU group showed delay of a- and b-waves at week 2 (p = 0.008, 0.008, respectively), and showed decreased amplitudes of the a- and b wave at week 2 (p = 0.008, 0.021, respectively), and there was no significant change at week 6 or 12 compared with the results of week 2. (**n**) The flicker amplitude in the 10 mg/mL MNU group showed a decrease in amplitude at week 2 (p = 0.008) and then remained decreased. *ffERG* full field electroretinogram, *MNU N*-methyl-*N*-nitrosourea, *DA *dark adaptive, *LA *light adaptive.

The 5 mg/mL MNU group included 9 cases of moderate degeneration and 1 case of severe degeneration. The mfERG of moderate degeneration cases produced severely decreased amplitudes and focal noise signals in the ffERG field. The delay of b-wave in DA 0.01 ffERG was not statistically significant at week 2 and at week 6. The mean amplitude of b-waves in the DA 0.01 ffERG showed a significant decrease at week 2, but no difference was evident between weeks 2 and 6. The mean implicit time in DA 10.0 ffERG showed a temporal delay at week 2. The mean amplitude in the DA 10.0 ffERG decreased at week 2 and then remained low from week 2 to week 6. The mean implicit time in LA 3.0 ffERG was not changed at weeks 2 and 6. The mean amplitude in LA 3.0 ffERG showed a decrease at week 2 and remained low at week 6. The mean amplitude of LA 3.0 flicker showed a decrease at week 2 and remained low at week 6 (Fig. 5, Table 1).

**Table 1:** The ffERG findings of the 5 mg/mL MNU group (n = 10).

	Baseline	Week 2	Week 6	p-value between baseline and Week 2	p-value between Week 2 and Week 6	p-value between baseline and Week 6
Mean implicit time of b-wave in the DA 0.01 (ms)	75.68 ± 13.72	85.30 ± 29.64	77.50 ± 15.05	0.028^a^	0.866^a^	0.176^a^
Mean amplitude of b-wave in the DA 0.01 (µV)	55.61 ± 5.73	8.34 ± 5.73	9.19 ± 7.69	0.028^a^	0.575^a^	0.013^a^
Mean implicit time of a-wave in DA 10.0 (ms)	16.55 ± 1.98	20.05 ± 2.08	19.35 ± 3.59	0.028^a^	0.612^a^	0.086^a^
Mean implicit time of b-wave in DA 10.0 (ms)	40.75 ± 5.26	64.07 ± 16.44	50.36 ± 5.01	0.018^a^	0.018^a^	0.005^a^
Mean amplitude of a-wave in the DA 10.0 (µV)	52.26 ± 17.57	16.78 ± 19.06	11.41 ± 5.56	0.005^a^	0.499^a^	0.005^a^
Mean amplitude of b-wave in the DA 10.0 (µV)	94.94 ± 35.48	39.92 ± 39.92	26.09 ± 18.83	0.005^a^	0.398^a^	0.005^a^
Mean implicit time of a-wave in LA 3.0 (ms)	14.72 ± 2.32	23.01 ± 8.27	17.27 ± 1.93	0.062^a^	0.090^a^	0.066^a^
Mean implicit time of b-wave in LA 3.0 (ms)	35.98 ± 5.73	50.95 ± 9.04	35.87 ± 2.86	0.063^a^	0.018^a^	0.678^a^
Mean amplitude of a-wave in LA 3.0 (µV)	24.15 ± 15.93	4.03 ± 1.86	4.79 ± 2.95	0.018^a^	1^a^	0.005^a^
Mean amplitude of b-wave in LA 3.0 (µV)	114.52 ± 59.94	24.96 ± 19.36	24.96 ± 15.57	0.018^a^	1^a^	0.007^a^
Mean amplitude of LA 3.0 flicker (µV)	94.16 ± 73.74	11.79 ± 9.18	18.41 ± 12.02	0.028^a^	0.237^a^	0.017^a^

*DA *dark adaptive, *LA *light adaptive.

^a^Wilcoxon-signed ranked test.

#### Fundus infrared reflectance and spectral-domain optical coherence tomography

**Figure 6:**
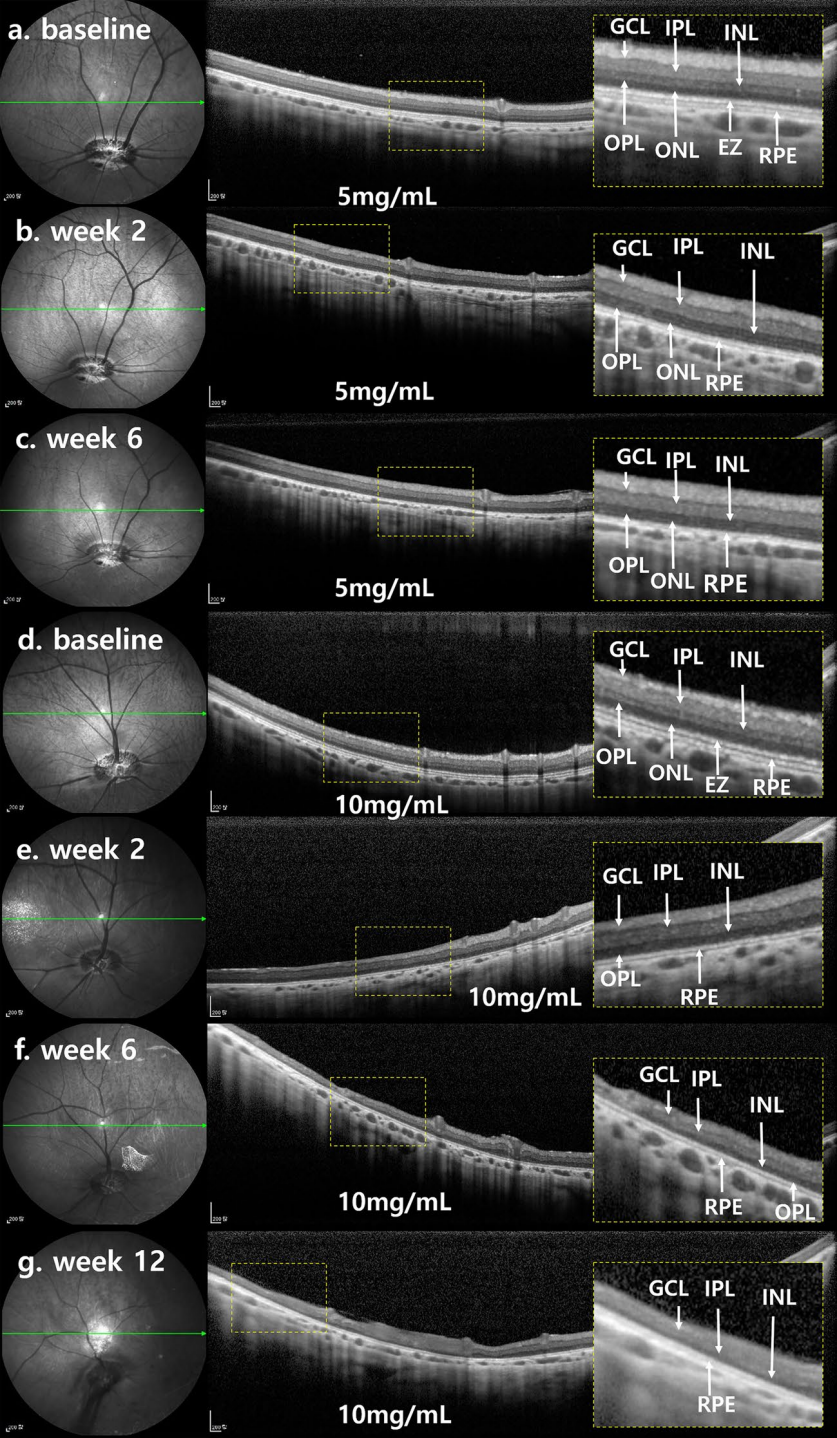
SD-OCT finding of each representative case of 5 mg/mL MNU and 10 mg/mL MNU. (**a–g**) Each magnified SD-OCT image of the case is from the right side and shown with a dashed-line box. (**a**–**c**). OCT images of a representative case with moderate outer degeneration in the 5 mg/mL group. (**a**,**b**) OCT images at week 2 showed intact GCL and RPE. The EZ was indistinguishable at week 2. In addition, there was a decrease in OPL thickness. (**c**) OCT images remained stable at week 6 compared with the results of week 2. (**d–g**) OCT images of a representative case with severe outer degeneration in 10 mg/mL group. (**d**,**e**) On the OCT, unlike baseline, the ellipsoid zone was indistinguishable at week 2. In addition, there was a decrease in INL thickness and ONL. (**f**,**g**) OCT image at weeks 6 and 12 showed no significant change compared with the results of week 2. However, the signal intensity of OCT decreased at week 12 due to anterior capsular opacity. *SD-OCT *spectral domain optical coherence tomography, *GCL *ganglion cell layer, *IPL *inner plexiform layer, *INL *inner nuclear layer, *OPL *outer plexiform layer, *ONL *outer nuclear layer, *EZ *ellipsoid zone, *RPE *retinal pigment epithelium.

**Figure 7:**
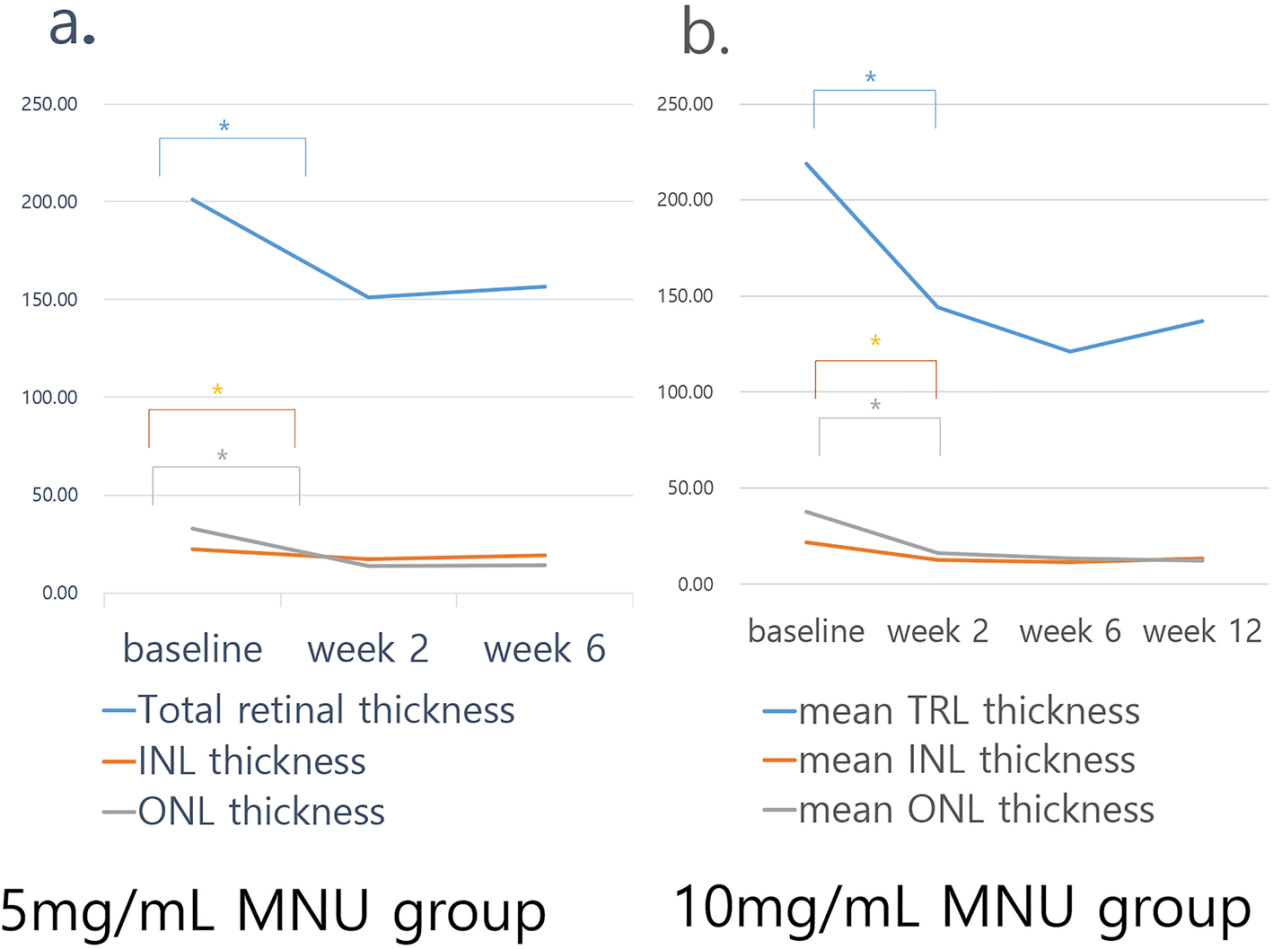
Sequential changes of total retinal thickness, inner nuclear layer thickness, and outer nuclear layer thickness. (**a**) In the 5 mg/mL MNU group, there was a significant decrease in INL, ONL, and total retinal thickness in week 2. After week 2, the subsequent changes were minimal and remained so until week 6. (**b**) In a 10 mg/mL MNU group, there was also a significant decrease of INL, ONL, and total retinal thickness at week 2. After week 2, the subsequent changes were minimal and remained decreased through week 12. *TRL *total retinal thickness, *INL *inner nuclear layer, *ONL *outer nuclear layer, *MNU N*-methyl-*N*-nitrosourea).

SD-OCT images at week 2 showed intact GCL and RPE. The ellipsoid zone on 55-degree SD-OCT fields was barely distinguishable at week 2 (Fig. 6), and outer plexiform layer thickness decreased. From week 2 to week 6, subsequent changes were minimal and similar among layers. Mean thicknesses of INL, ONL, and TRL showed significant decrease at week 2. From week 2 to week 6, changes were minimal (Fig. 7, Table 2).

**Table 2:** Optical coherence tomography findings of the 5 mg/mL MNU group (n = 10) and 10 mg/mL MNU groups (n = 13).

	Baseline	Week 2	Week 6 (12)^a^	p-value between baseline and Week 2	p-value between Week 2 and Week 6 (12)^a^	p-value between baseline and Week 6 (12)^a^
Mean INL thickness in 5 mg/mL MNU group (μm)	22.38 ± 4.93	17.46 ± 7.58	19.41 ± 3.89	< 0.001^b^	0.831^b^	< 0.001^b^
Mean ONL thickness in 5 mg/mL MNU group (μm)	32.93 ± 9.56	13.92 ± 7.31	14.44 ± 5.69	< 0.001^b^	0.575^b^	< 0.001^b^
Mean TR thickness in 5 mg/mL MNU group (μm)	201.10 ± 24.95	151.20 ± 38.11	156.74 ± 15.01	< 0.001^b^	0.136^b^	< 0.001^b^
Mean INL thickness in 10 mg/mL MNU group (μm)	21.80 ± 5.81	12.80 ± 2.78	12.34 ± 3.40	< 0.001^b^	0.831^b^	< 0.001^b^
Mean ONL thickness (μm) in 10 mg/mL MNU group (μm)	37.93 ± 6.13	16.20 ± 8.35	12.15 ± 8.97	< 0.001^b^	0.575^b^	< 0.001^b^
Mean TR thickness in 10 mg/mL MNU group (μm)	201.10 ± 24.95	144.00 ± 34.88	136.77 ± 43.39	< 0.001^b^	0.136^b^	< 0.001^b^

*INL *inner nuclear layer, *MNU N*-methyl-*N*-nitrosourea, *ONL *outer nuclear layer, *POD *postoperative day, *TR *total retinal thickness.

^a^At week 12 in 10 mg/mL MNU group.

^b^Wilcoxon-signed ranked test.

#### Hematoxylin and eosin and immunohistochemistry

**Figure 8:**
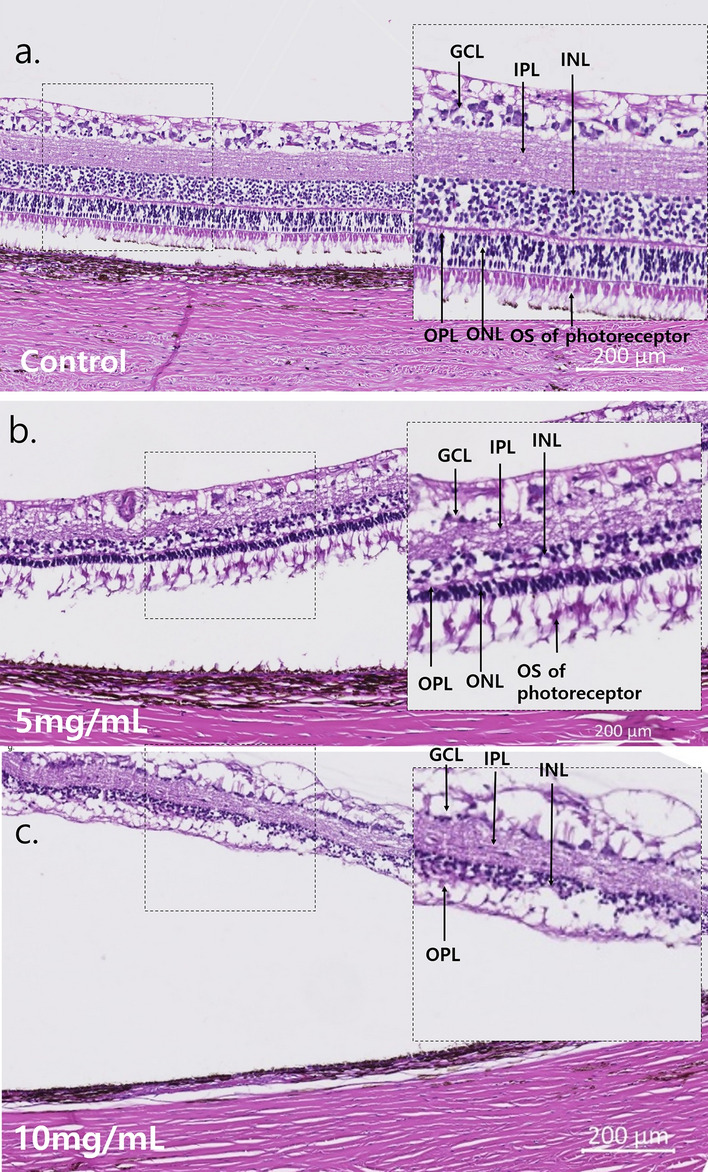
H&E staining of a representative case of the 5 mg/mL MNU and 10 mg/mL MNU groups. (**a–c**) Each magnified H&E staining image was from the right side and is shown with a dashed-line box. (**a**) H&E staining of a control case. Each count number of ONL, INL, and GCL in dotted enlarged area was 201, 307, and 49, respectively. (**b**) H&E staining of a representative case with moderate outer degeneration in the 5 mg/mL group. In H&E staining, all the INL, GCL and NFL were intact, but the density of INL and GCL decreased. Each count number of ONL, INL, and GCL in dotted enlarged area was 100, 91, and 16, respectively. (**c**) H&E staining of a representative case with severe outer degeneration in the 10 mg/mL group. ONL cells were rarely observed among INL or combined INL with ONL cells. Each count number of ONL, INL, and GCL in dotted enlarged area was 0, 112, and 29, respectively. *H&E *hematoxylin and eosin, *GCL *ganglion cell layer, *IPL *inner plexiform layer, *INL *inner nuclear layer, *OPL *outer plexiform layer, *ONL *outer nuclear layer, *OS *outer segment, *RPE *retinal pigment epithelium.

**Figure 9:**
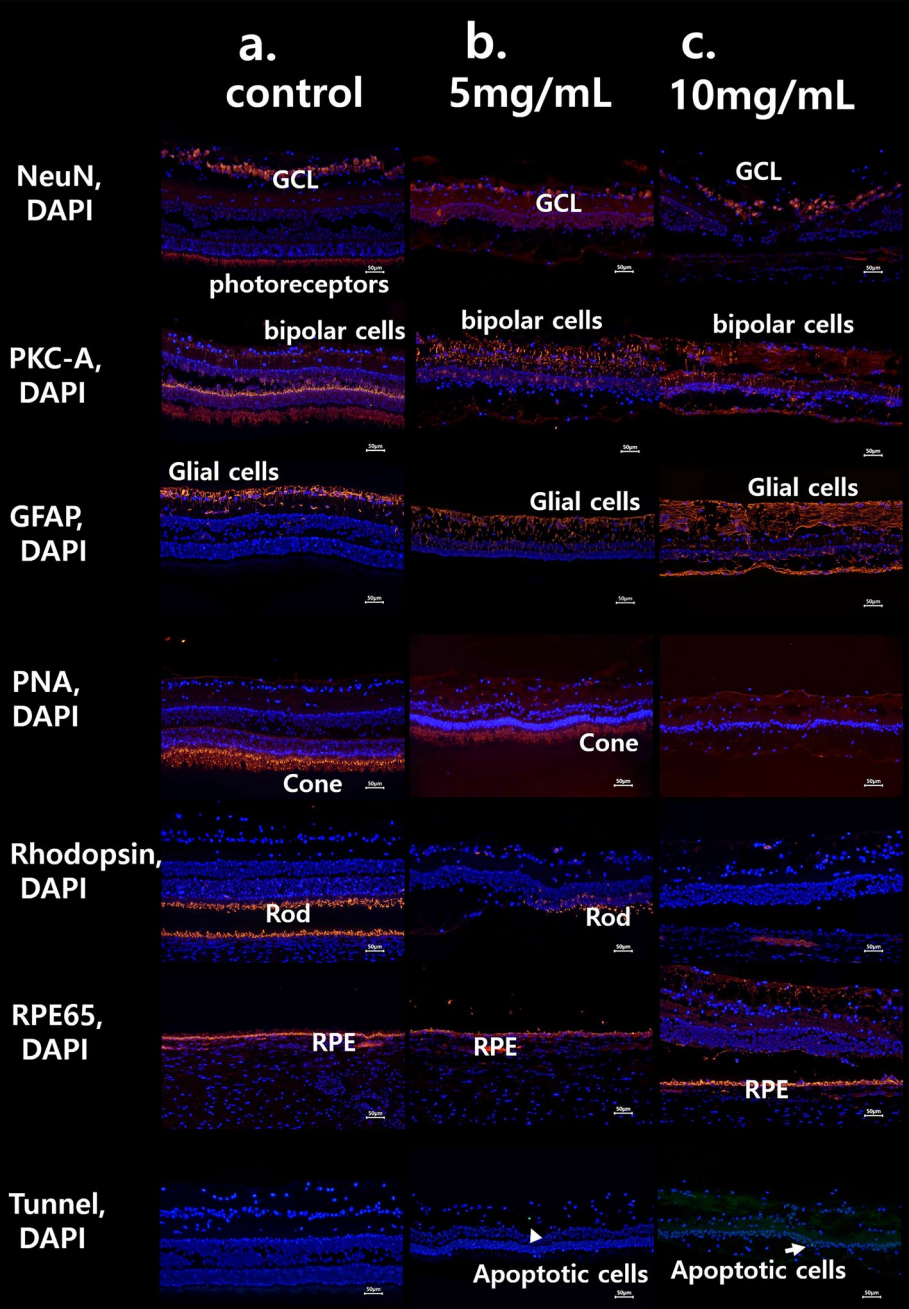
The immunohistochemistry findings of each representative case of 5 mg/mL MNU group and 10 mg/mL MNU group. (**a**) The normal immunohistochemistry of a control case. Rhodopsin staining and PNA staining showed well-stained rod and cone cells (each percentage of the stained layer = 15.22% and 14.86%). (**b**) The immunohistochemistry of a representative case with moderate outer degeneration in the 5 mg/mL group. NeuN staining showed intact ganglion cells and the co-staining of photoreceptors. PKC-α staining showed intact bipolar cells, and GFAP staining showed nearly normal muller cells. Rhodopsin staining showed severely decreased rod cells, and PNA staining showed focal intact cone cells (each percentage of the stained layer = 4.11% and 14.15%). RPE65 staining showed intact RPE and rare co-staining of photoreceptors. There was no apoptotic cell in Tunnel staining. (**c**) The immunohistochemistry of a representative case with severe outer degeneration in 10 mg/mL group. NeuN and PKC-α stainings showed intact ganglion cells and bipolar cells, respectively. There was no co-staining of photoreceptors in NeuN staining. GFAP staining showed increased staining, especially in the outer retina. Rhodopsin and PNA staining showed relatively rare stained lesions of rod and cone cells (each percentage of the stained layer = 0% and 0%) with co-staining of RPE cells. RPE 65 staining showed intact RPE cells and Tunnel staining showed no stained lesions. *RPE *retinal pigment epithelium, *GCL *ganglion cell layer, *IPL *inner plexiform layer. Each percentage of the stained layers was evaluated by the stained area/all layer area using ImageJ software (1.53a version, National Institutes of Health, Bethesda, MD, USA).

In H&E staining of moderate degeneration cases (n = 9), the INL, GCL, and NFL were nearly intact (Fig. 8). Rhodopsin staining showed severely decreased rod cells, and PNA staining showed focal intact cone cells. RPE65 staining showed intact RPE and rare co-staining of photoreceptors. NeuN staining showed intact ganglion cells and co-staining of few photoreceptors. PKC-α staining showed intact bipolar cells, and GFAP staining showed nearly normal Müller cells. No apoptotic cells were evident in Tunel staining (Fig. 9).

### Efficacy experiment results of the 10 mg/mL MNU group (n = 13)

#### Full-field electroretinography and multifocal electroretinography

The 10 mg/mL MNU group included 11 cases of severe degeneration and 2 cases of moderate degeneration. A significant delay in implicit time was recorded in the DA 0.01 ffERG at week 2, but the change was not statistically significant at weeks 6 or 12. The amplitude of b-waves in the DA 0.01 ffERG showed a significant decrease at week 2. In DA 10.0 ffERG, there was a delay of implicit time at week 2. The amplitudes on DA 10.0 ffERG decreased at week 2. In the LA 3.0 ffERG, decreased amplitude was observed at week 2. Above each amplitude and implicit time remained decreased and delayed from week 2 to week 12, respectivley. The implicit time in LA 3.0 ffERG did not change at week 2, and there was no significant change from week 2 to week 12. The amplitude of LA 3.0 flicker showed a decrease at week 2 and remained decreased from week 2 to week 12. In the mfERG of all cases, only noise signals were observed at each follow-up period (Fig. 5, Table 3).

**Table 3:** The ffERG findings of the 10 mg/mL MNU group (n = 13).

	Baseline	Week 2	Week 12	p-value between baseline and Week 2	p-value between Week 2 and Week 12	p-value between baseline and Week 12
Mean implicit time of b wave in the DA 0.01 (ms)	70.90 ± 15.34	88.21 ± 33.44	74.25 ± 25.68	0.260^a^	0.116^a^	0.241^a^
Mean amplitude of b wave in the DA 0.01 (µV)	71.24 ± 39.68	4.79 ± 6.59	3.86 ± 4.09	0.008^a^	0.917^a^	0.005^a^
Mean implicit time of a wave in In DA 10.0 (ms)	16.61 ± 2.66	34.36 ± 22.01	27.09 ± 12.78	0.008^a^	0.293^a^	0.037^a^
Mean implicit time of b wave in In DA 10.0 (ms)	39.94 ± 4.42	71.11 ± 25.79	63.41 ± 19.83	0.008^a^	0.249^a^	0.007^a^
Mean amplitude of a wave in the DA 10.0 (µV)	54.62 ± 19.24	7.58 ± 7.53	4.69 ± 2.74	0.008^a^	0.917^a^	0.005^a^
Mean amplitude of b wave in the DA 10.0 (µV)	102.89 ± 46.66	13.79 ± 20.94	4.36 ± 5.09	0.008^a^	0.116^a^	0.005^a^
Mean implicit time of a wave in LA 3.0 (ms)	12.83 ± 4.05	20.88 ± 4.33	28.76 ± 18.15	0.011^a^	0.345^a^	0.005^a^
Mean implicit time of b wave in LA 3.0 (ms)	65.89 ± 11.29	102.38 ± 24.70	85.38 ± 19.42	0.008^a^	0.249^a^	0.005^a^
Mean amplitude of a wave in LA 3.0 (µV)	24.89 ± 11.99	3.11 ± 4.22	1.49 ± 0.91	0.008^a^	0.917^a^	0.013^a^
Mean amplitude of b wave in LA 3.0 (µV)	137.42 ± 68.11	16.65 ± 23.43	3.96 ± 3.64	0.021^a^	0.917^a^	0.017^a^
Mean amplitude of LA 3.0 flicker (µV)	107.14 ± 63.64	8.19 ± 14.56	4.08 ± 3.09	0.008^a^	0.917^a^	0.005^a^

*DA *dark adaptive, *LA *light adaptive.

^a^Wilcoxon-signed ranked test.

#### Fundus infrared reflectance and spectral-domain optical coherence tomography

On SD-OCT, the EZ was indistinguishable at week 2. The thicknesses of INL, ONL, and TRL appeared to decrease at week 2 (Fig. 6). Further losses of INL and ONL were not detected at week 6 or 12. In addition, there was decrease in mean thicknesses of INL, ONL, and TRL. From week 2 to week 12, each mean thickness showed no subsequent changes (Fig. 7, Table 2).

#### Hematoxylin and eosin and immunohistochemistry

In H&E staining of severe degeneration cases (n = 11), ONL cells were rarely observed among mainly INL (Fig. 8). Rhodopsin and PNA staining showed no rod or cone cells with co-staining of RPE cells. RPE 65, PKC-α, and NeuN staining showed intact RPE cells, bipolar cells, and ganglion cells, respectively. There was no co-staining of photoreceptors in the NeuN stain. GFAP staining showed increased staining, especially in the outer retina, and Tunel staining showed no stained lesions (Fig. 9).

## Discussion

Our study used a mini-pig model to evaluate the effect of MNU on photoreceptors because the size, vascular anatomy, vascular function, and immunology of the pig eye are comparable to those of a human eye^25–28^. Although the pig retina does not have a fovea, a so-called visual streak of high cone density (rod:cone ratio = 3:1 [central], 8:1 [mean], and 16:1 [peripheral]) is located in a horizontal axis near the posterior pole of the fundus and is similar to that of human fovea^29,30^. Therefore, for preclinical studies, many pig disease models have been studied^12,13,19,31–34^. Noel et al. showed that concentration-dependent reduction in both ffERG b-wave and mfERG N1–P1 amplitudes compared to baseline after intravenous Iodoacetic acid injection. The fundus of pig treated with ≥ 10 mg/kg Iodoacetic acid was abnormal with thinner retinal vessels and pale optic discs like retinitis pigmentosa, but there was no extinction of photopic ffERG^19^. Geographic atrophy in pig was successfully induced by subretinal 0.01 mg/mL SI injection^31^. Diabetic retinopathy pig models showed several similar signs of diabetic retinopathy like those seen in humans^32,33^. Kleinwort et al. showed that retinas from INS C94Y pigs exposed to hyperglycemia for more than 2 years have intraretinal microvascular abnormalities and central retinal edema in a cone-riched region^33^. Because of its similarity with human, the pig retina might be considered as enough model for glaucoma studies. One study showed that chronically increased intraocular pressure could induce retinal ganglion cell death significantly in the mid-peripheral and peripheral retina, in which the temporal quadrants were mainly damaged^34^.

Although rodent eyes could serve as an alternative to pig retinas in spite of their small size because mice experience similar retinal vascular development and retinal histological structures, the rodent retina is predominantly composed of rod cells (rod:cone ratio = approximately 98:2)^35^. A rabbit’s eyeball size is similar to that of humans, but rabbit retinal vascular development differs from that of the human eye (melanotic vs. holangiotic)^36,37^. Numerous studies have employed pigs for large-animal models in the study of retinal diseases and therapeutic interventions^38,39^.

We analyzed the effectiveness of temporal MNU tamponade (exposure) with vitrectomy to induce unilateral diffuse outer retinal degeneration in mini-pig. In a previous experiment, globally diffuse outer retinal degeneration using intravitreal MNU injection was not obtained even after vitrectomy. Instead, localized outer retinal degeneration was found in the gravitational direction after intravitreal MNU injection in vitrectomized eyes^23^. The water solubility of MNU is 14.4 g/L (14.4 mg/mL) at 24 °C, but a small amount of intravitreal MNU injection (0.05 mL) appeared to be localized on the retinal surface by gravity. Because MNU is sensitive to moisture and light and hydrolyzes in water with a half-life of 1.2 h at pH 7 at 20 °C^40–42^, most effects on the retina were thought to take place rapidly. This may explain why small intravitreal MNU injections have a spatially localized effect on the retina. To avoid this problem, we temporarily filled a whole vitreous cavity with MNU solution diluted in BSS. The MNU tamponade was performed for 10 min, which was sufficient for retina whitening (Supplementary Video 1). After 10 min, as much as possible of the MNU solution was removed by 2 or 3 cycles of air-fluid exchange, and BSS tamponade was performed in the vitreous cavity at the end of surgery.

Degree of degeneration varied according to MNU concentration. In a preliminary experiment, degree of outer retinal degeneration of the retina increased with MNU concentration. Temporal tamponade of 5 mg/mL MNU and 10 mg/mL MNU during vitrectomy was effective to induce respective moderate and severe outer retinal degeneration without retinal atrophic change. Zulliger et al. showed retinal changes to be MNU concentration-dependent, a relationship that was most obvious with the ONL^43^. Other reports showed a dose-dependent response over the threshold dose as a carcinogen^44,45^.

Two moderate retinal degeneration cases in the 10 mg MNU group showed severe outer retinal change in most of the area except the far peripheral retina, where the retinal structure was intact. That may explain why moderate retinal degeneration cases presented diminished amplitudes in ffERG despite ONL layer loss in the 55-degree SD-OCT field. Remnant peripheral vitreous fluid may block adequate exposure of MNU to the far peripheral retina, which could be predicted after MNU solution tamponade during vitrectomy. After 10 min of MNU solution tamponade, the color of the degenerated retina changed to white; this color change was not evident in the non-degenerated far periphery during vitrectomy (Supplementary Video 1). This suggests that posterior vitreous detachment at the far periphery is important to ensure sufficient application of MNU to the whole retinal area.

Most degenerative changes were observed at week 2, and no further progress was observed during the study period. Our immunochemistry results showed rare apoptotic cells in Tunnel stain at week 6 or week 12. One study using intraperitoneal MNU injection reported that MNU induces peak photoreceptor cell loss 3 days post-injection during a 7-day follow-up period^46^. Numerous bone marrow–derived cells migrated to the retina and differentiated into immunoreactive microglia in response to photoreceptor degeneration within 7 days of MNU injection^47^. In the apoptosis cascade, down-regulation of Bcl-2 and up-regulation of Bax were seen at 12 h, and caspase-family activity peaked 72 h after MNU treatment^48^.

Our study has some limitations. First, only 6 weeks of follow-up were conducted in the 5 mg/mL MNU groups, and the degree of outer retinal degeneration could worsen after 6 weeks. There were no significant changes from week 2 to week 6 in the 5 mg/mL MNU group. No significant changes were also observed from week 2 to week 12 in the 10 mg/mL MNU group. Furthermore, there were rare apoptotic cells of Tunnel stain in both groups at week 6 or 12. Because no evidence of retinal degeneration progress was observed in the preliminary study, the relatively short follow-up period for the 5 mg/mL MNU group compared with the 10 mg/mL group did not appear to affect the results significantly. Second, the unstable chemical properties of MNU in water may have compromised the reliability and consistency of the results.

In conclusion, temporal exposure of MNU induced outer retinal degeneration in vitrectomized pig eyes. The degree of retinal degeneration was modulated by adjusting MNU concentration. The MNU-induced pig model used in this study is a candidate for further research.

## Materials and methods

### Animals

All procedures adhered to the Association for Research in Vision and Ophthalmology Statement for the Use of Animals in Ophthalmic and Vision Research. Approval for this study was obtained from the Institutional Animal Care and Use Committee of Korea University College of Medicine (KOREA-2018-0002-C1).

In the preliminary experiment, a total of 5 eyes from 5 female mini-pigs (MICROPIG, APURES Co., Ltd, Pyeongtaek-si, Korea) were used to determine degree of retinal change according to MNU concentration (4, 8, 10, 12, and 16 mg/mL). Each concentration was tested in 1 mini-pig. To identify morphological changes in the retina, fundus infrared reflectance (IR) images, 55-degree spectral-domain optical coherence tomography (OCT), full-field electroretinography (ffERG), and multifocal ERG (mfERG) were performed at baseline and 2, 6, and 12 weeks after surgery. Histological examinations using hematoxylin and eosin (H&E) and immunohistochemistry staining were performed on selected mini-pig eyes 12 weeks after surgery. In the second efficacy experiments, which were based on the first experiment, the effect of 5 mg/mL MNU (n = 10) was evaluated in the mini-pigs following the protocol used in the preliminary experiment, with the exception of week 12 follow-up examinations. Further study with 10 mg/mL MNU (n = 13) followed the same protocol with week 12 follow-up examinations.

### Vitrectomy with MNU loading

The mini-pigs were anesthetized by intravenous injection of alfaxalone (1 mg/kg; Alfaxan, Vetoquinol, West Sussex, UK) into the marginal auricular vein following premedication, which comprised a subcutaneous injection of atropine (0.05 mg/kg), intramuscular injection of xylazine (1 mg/kg; Rompun, Bayer Corp., Shawnee Mission, KA, USA), and azaperone (4 mg/kg; Stresnil, Mallinckrodt Veterinary Inc, Indianapolis, IN, USA). After general anesthesia, the eye was irrigated with 5% povidone-iodine and draped for surgery. Three-port, 23-gauge vitrectomy (Associate; DORC, Zuidland, Netherlands) was performed with an indirect lens (Oculus BIOM ready, Oculus Surgical, Inc., FL, USA). Three ports were prepared by inserting trocar cannulas into the sclera 3 mm from the limbus at ventromedial, ventrolateral, and dorsomedial sides. The vitreous was removed using a vitreous cutter, while continually supplying balanced salt solution (BSS; Alcon, Fort Worth, TX). After core vitrectomy, posterior vitreous detachment was induced gently to avoid an iatrogenic retinal break. Both peripheral vitrectomy and lensectomy were performed. Air-fluid exchange was then carried out^23,24^. The vitreous cavity was fully tamponaded with different concentrations of MNU solution (Oakwood Products Inc., West Columbia, SC, USA) for 10 min, and MNU solution was removed by air-fluid exchange. Finally, the vitreous cavity was rinsed 3 times and filled with BSS to ensure complete removal of the MNU (Supplementary Video 2).

### Full-field electroretinography and multifocal electroretinography

The ffERG protocol was based on the international standard for electroretinography from the International Society for Clinical Electrophysiology of Vision (ISCEV)^49,50^. The mini-pigs were anesthetized as described above and dark-adapted for 30 min, after which the pupils were dilated. Light stimulation and ffERG signal recording were performed with a commercial system (RETIcom; Ronald Consult, Germany), using a contact lens electrode with a built-in light resource (Kooijman/Damhof ERG lens; Medical Workshop BV, Netherlands). The reference and ground electrodes were subdermal platinum needle electrodes. Reference electrode was placed in the skin near the lateral canthus of the eyes, and a ground electrode was placed on the forehead between the two eyes. The mfERG protocol was based on the ISCEV standard^50,51^. Light stimulation at a distance of 26 cm from a 19-inch liquid crystal display monitor and mfERG signal recording used a commercial system (RETIpot; Ronald Consult, Germany). The contact lens electrode used the same instruments as in ffERG.

The retinal degeneration was classified according to the amplitudes of a dark adaptive (DA) 10.0 a-wave amplitude. The degree of moderate outer retinal degeneration was defined as DA 10.0 a-wave amplitude between ≥ 10% and < 60% of baseline amplitude. The degree of severe degeneration was defined as DA 10.0 a-wave amplitude < 10% of baseline amplitude including a flat signal or noise with no a or b wave on DA 0.01, 10, light adaptive (LA) 3.0 ffERG.

### Fundus infrared reflectance and spectral-domain optical coherence tomography

**Figure 10:**
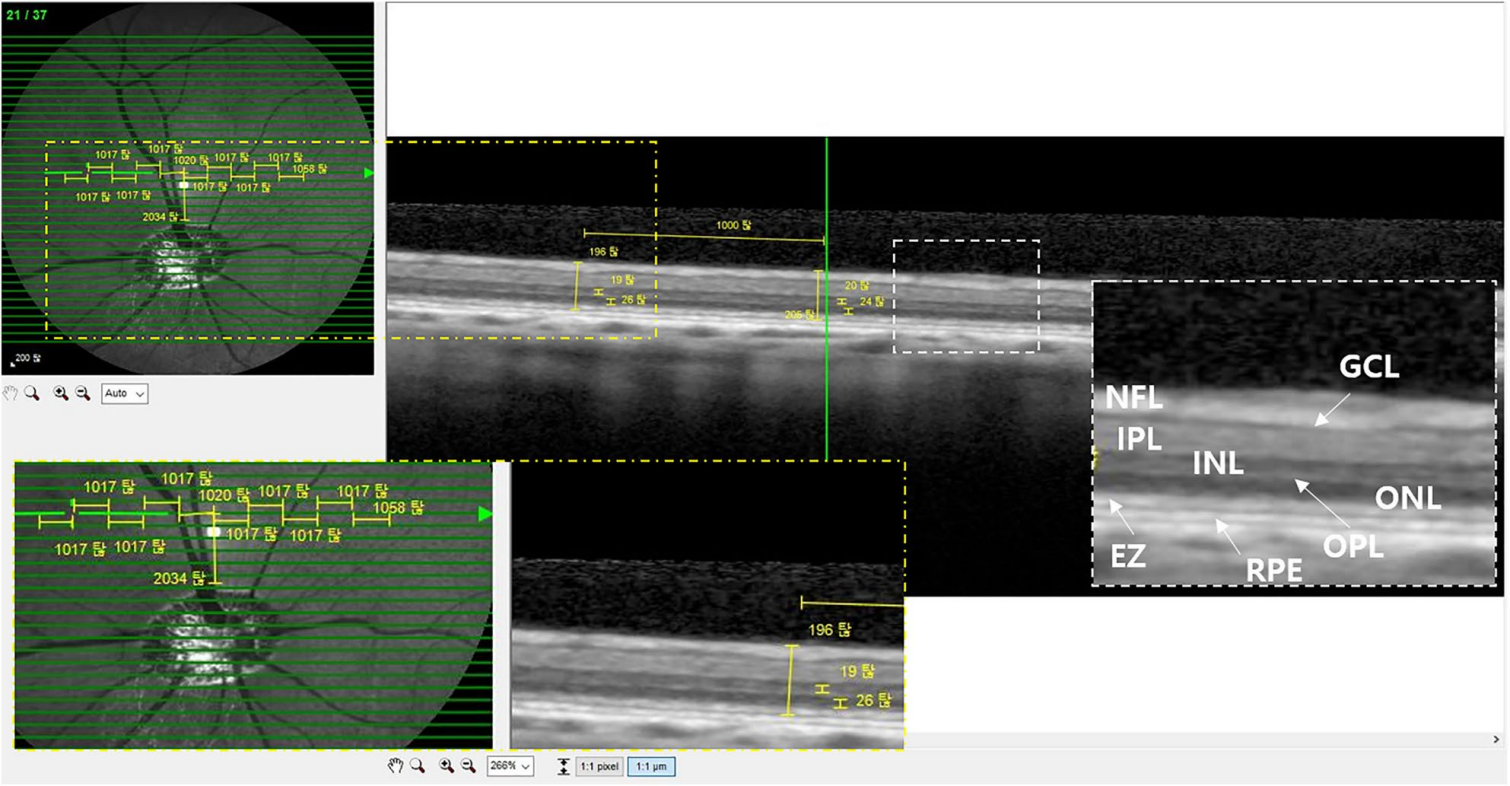
Measurement of total retinal thickness, inner nuclear layer thickness, and outer retinal thickness. Total retinal thickness, inner nuclear layer thickness, and outer retinal thickness were measured at 10 points with 1 mm intervals on the visual streak. Magnified SD-OCT images are shown with a white or yellow dashed-line box. Magnified SD-OCT images in yellow dashed-line boxes show estimated total retina thickness, inner nuclear thickness, and outer retinal thickness. Magnified SD-OCT images in white dashed-line boxes show NFL, GCL, IPL, INL, OPL, EZ, and PRE. *NFL *nerve fiber layer, *GCL *ganglion cell layer, *IPL *inner plexiform layer, *INL *inner nuclear layer, *OPL *outer plexiform layer, *EZ *ellipsoid zone, *RPE *retinal pigment epithelium.

A-scan biometry (SW-1000, Suoer, China) was obtained to measure the axial length of the eyeball at baseline. Images were obtained with 55-degree fundus infrared reflectance (IR) and spectral-domain optical coherence tomography (SD-OCT) images using the Spectralis OCT system (Heidelberg Engineering GmbH, Heidelberg, Germany). Vertical and horizontal line scans, as well as raster scans (33 B-scans over a 16.5 × 16.5-mm area in a 55-degree image), were performed at high resolution (1536 A-scans per B-scan, lateral resolution = 10 µm/pixel in 55-degree image). Up to 100 images were averaged in automatic real-time mode to obtain a high-quality mean image. Total retinal layer (TRL) thickness was measured along a horizontal line perpendicular to the retinal layers in cross-sectional images. Inner nuclear layer (INL) thickness and outer nuclear layer (ONL) thickness were measured at 10 different areas, along with a horizontal visual streak 2 mm dorsal to the optic disc (Fig. 10). TRL thickness was defined as the distance between the inner margin of the internal limiting membrane to the inner margin of the retinal pigment epithelium (RPE) layer. INL thickness was defined as the distance between the outer margin of the inner plexiform layer to the inner margin of the outer plexiform layer. ONL thickness was defined as the distance between the outer margin of the outer plexiform layer to the inner margin of the ellipsoid zone (EZ)^52^.

### Histological examination

Immediately after euthanasia, the subject eyes were enucleated, immersion-fixed in Davidson’s solution for 24 h, dehydrated, and embedded in paraffin. Sections 4 µm in width were cut and stained with H&E. The slides were examined for retinal pathological changes using a light microscope (BX-53, Olympus Corp., Tokyo, Japan) and photographed with a slide scanner (Zeiss Axio Scan. Z1, White Plains, NY, USA).

### Immunohistochemistry

Tissue sections were deparaffinized*, *rehydrated, and microwave-heated in antigen retrieval buffer (tris-ethylenediaminetetraacetic acid solution, pH 9.0). Sections were then blocked with 4% horse serum in phosphate-buffered saline with Tween, followed by incubation with each primary antibody at 4 °C overnight. Nuclei were counterstained with 4,6-diamidino-2-phenylindole (Sigma-Aldrich, St. Louis, MO, USA), and Alexa Fluor 594-conjugated goat anti-mouse secondary antibody (1:300; Biogend, Taipei City, Taiwan) was used to perform fluorescence detection for anti-PKC-α (1:200; Invitrogen, Carlsbad, CA, USA) staining, anti-GFAP (1:300; NOVUSBIO, Centennial, CO, USA) immunostaining, anti-NeuN (1:250; Merck, Darmstadt, Land Hessen, Germany) immunostaining, anti–peanut agglutin (PNA) (1:300; VECTOR, Woodinville, WA, USA) immunostaining, anti-rhodopsin (1:200; Rockland, Limerick, PA, USA) immunostaining, and anti-RPE65 (1:200; Invitrogen, Carlsbad, CA, USA) immunostaining (Table 4). Cells stained by TUNEL were evaluated using fluorescence microscopy (T2000-U; Nikon, Tokyo, Japan).

**Table 4:** Summary of immunohistochemistry for pig retinal degeneration model.

	Sequence of antibody (primary/secondary)	Dilution	Industry	Targeted cells or layers
Anti-PKC-α	Primary	1:200	Invitrogen	(rod) Bipolar cells. Targeted protein kinase (PKC)-α is abundant in retinal bipolar cells
Anti-GFAP	Primary	1:300	NOVUSBIO	Müller cells, astrocytes, and glical cells. Targeted GFAP is a member of the class III intermediate filament protein family abundant in astrocytes and glial cells
Anti-NeuN	Primary	1:250	Merck	Retinal ganglion cells. Targeted NeuN is DNA-binding, neuron-specific protein
Anti-PNA	Primary	1:300	VECTOR	Cone cells. Targeted S opsin exists in cone cells
Anti-rhodopsin	Primary	1:200	Rockland	Rod cells. Targeted retinal rhodopsin exists in rod cells
Anti-RPE65	Primary	1:200	Invitrogen	RPE cells. RPE65 is a major protein of the RPE
Alexa Fluor 594-conjugated goat anti-mouse	Secondary	1:300	Biogend	Capture or detection of mouse IgG in ELISA

*PKC-α *Protein kinase-α, *GFAP *Glial fibrillary acidic protein, *NeuN *Neuron-specific nuclear protein, *PNA *peanut agglutin, *RPE *retinal pigment epithelium, *IgG *Immunoglobulin G, *ELISA *Enzyme-linked immunosorbent assay.

### Statistical analysis

To compare the results from baseline to follow-up periods, Wilcoxon signed-rank test was used. Statistical analyses were done using SPSS version 21.0.0.0 (IBM, Armonk, NY, USA) software. All statics were two-tailed and p-values less than 0.05 were considered to be significant.

## Supplementary Information

Supplementary information 1.

Supplementary information 2.

Supplementary Figure 1.

Supplementary information 3.

Supplementary information 4.

## Data Availability

The datasets generated during and/or analyzed during the current study are available from the corresponding authors on reasonable request.
