# Supplementary figures and images for: An experimental pig model with outer retinal degeneration induced by temporary intravitreal loading of N-methyl-N-nitrosourea during vitrectomy
Source: Sci Rep. 2021 Jan 8;11:258. doi: 10.1038/s41598-020-79437-1 (PMC7794530; doi:10.1038/s41598-020-79437-1)

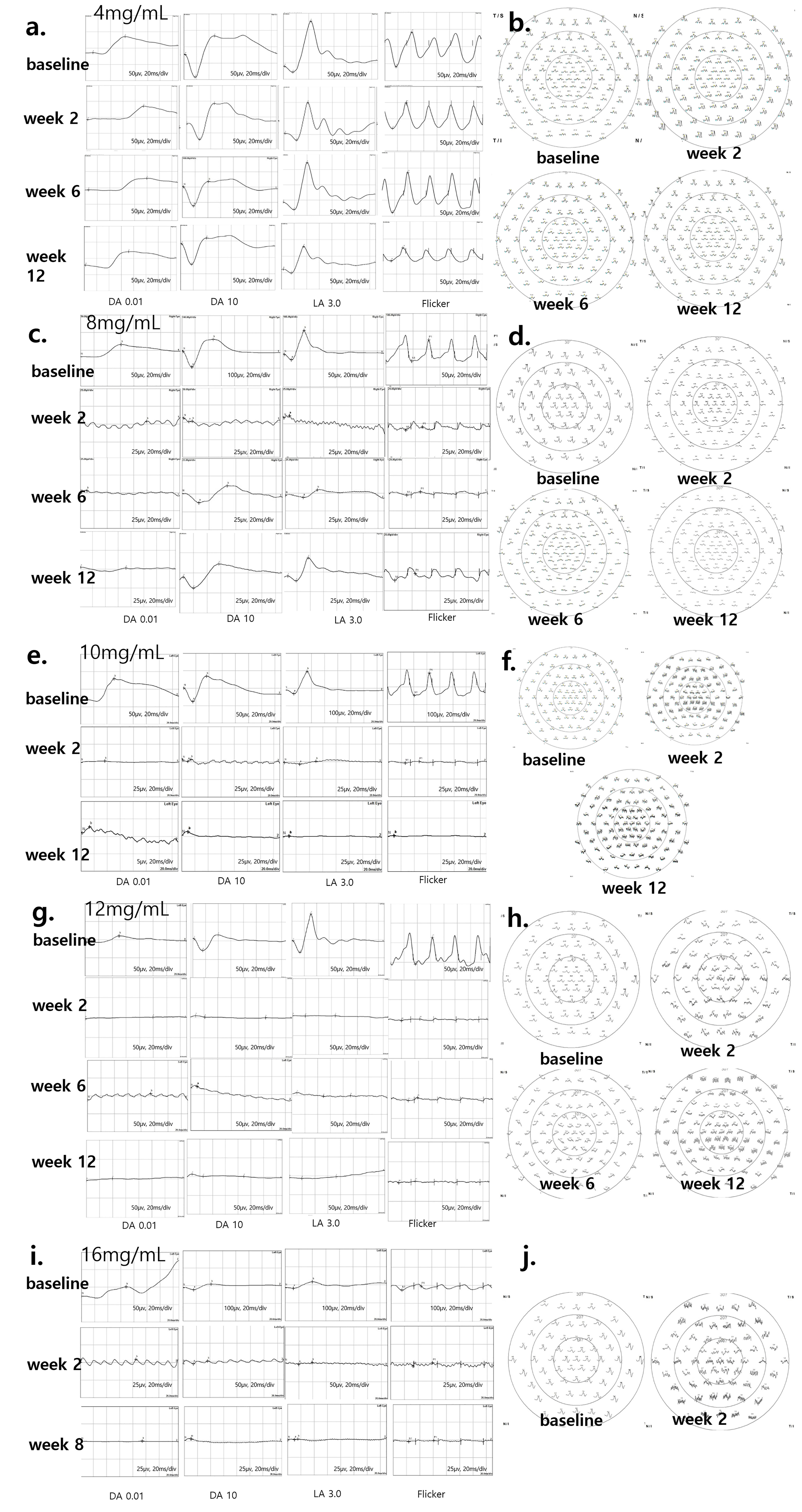

Supplement: Supplementary file 3 — Supplementary Figure 1. [file 41598_2020_79437_MOESM3_ESM.jpg]
