# Supplementary material for: An experimental pig model with outer retinal degeneration induced by temporary intravitreal loading of N-methyl-N-nitrosourea during vitrectomy
Source: Sci Rep. 2021 Jan 8;11:258. doi: 10.1038/s41598-020-79437-1 (PMC7794530; doi:10.1038/s41598-020-79437-1)
Supplement: Supplementary file 5 — Supplementary information 4. [file 41598_2020_79437_MOESM5_ESM.docx]

**An experimental pig model with outer retinal degeneration induced by temporary intravitreal loading of N-methyl-N-nitrosourea during vitrectomy**

Kwang-Eon Choi, MD,^1^ Vu Thi Que Anh, MD,^2^ Jee Taek Kim, MD, PhD, ^3^ Cheolmin Yun, MD, PhD,^1^ Seongkwang Cha,^4^ Jungryul Ahn,^4^ Yong Sook Goo, MD, PhD,^4^ Seong-Woo Kim, MD, PhD^1^

^1^ Department of Ophthalmology, Korea University College of Medicine, Seoul, Korea

^2^ Department of Ophthalmology, Hanoi Medical University, Hanoi, Vietnam

^3^ Department of Ophthalmology, Chung-Ang University College of Medicine, Seoul, Korea

^4^ Department of Physiology, Chungbuk National University College of Medicine, Cheongju, Korea

**Supplementary Figure and Video legends.**

Supplementary figure 1. ffERG findings of the retina in one case of each 4 mg/mL, 8 mg/mL, 10mg/mL, 12 mg/mL, and 16 mg/mL MNU from baseline to week 12. a, b, The 4 mg/mL MNU case. a. Scotopic and photopic ffERG results showed no significant change in implicit time or amplitude from baseline to weeks 2, 6, or 12. . b. mfERG in the 4 mg/mL MNU showed no signal flattening or noise signals at any follow-up period. c, d. The 8 mg/mL MNU case c. The amplitudes in scotopic and photopic ffERG decreased at week 2, but DA 10.0, LA 3.0, and Fliker waves were detected at week 6 and 12 with decreased amplitudes. d. mfERG in the 8 mg/mL MNU case showed focal noise signal and signal flattening from week 2 to week 12. e, f. The 10mg/mL MNU case. e. All signals of ffERG showed nearly flat down signals or noise signals from week 2 to week 12. f. The mfERG in a 10 mg/mL MNU case showed only noise signals at each follow-up period. g, h. The 12mg/mL MNU case. g. All signals of ffERG showed nearly flat down signals or noise signals from week 2 to week 12. h. The mfERG in a 12 mg/mL MNU case showed only noise signals at each follow-up period. i, j. Both ffERGs and mfERGs in a 16 mg/mL MNU case showed noise signals from week 2. (ffERG = full field electroretinogram; MNU = N-methyl-N-nitrosourea; DA = dark adaptive; mfERG = multifocal electroretinogram)

Supplementary Figure 2. SD-OCT findings of the retina in one case of each 4 mg/mL, 8 mg/mL, 10mg/mL, 12 mg/mL, and 16 mg/mL MNU at each follow up periods. a, b, c, d, e, f, g, h, i, j, k, l, m, n, o, p, q. Each magnified SD-OCT image of the case is on the right sided and shown with a dashed-line box. a, b, c, d. OCT image of the 4 mg/mL MNU case showed intact GCL, INL, ONL, photoreceptor layer, and RPE from baseline to week 12. e, f, g, h. The 8mg/mL MNU case. f. OCT image at week 2 showed an intact ganglion cell layer and retinal pigment epithelium. The boundary of the INL and ONL became indistinct compared with the SD-OCT image at baseline (e). The ellipsoid zone representing the photoreceptor layer became obscure. g, h. OCT images at weeks 6 and 12 weeks showed no change compared with that at week 2. i, j, k. The 10 mg/mL MNU case. j. On the OCT, unlike baseline (i), the ellipsoid zone, and outer nuclear layer (ONL) were indistinguishable at week 2. However, there was no significant decrease in INL thickness. k. The total retinal thickness of the 10 mg/mL MNU case at week 12 seemed to be focally thinner than at week 2. However, there was no significant decrease compared with the corresponding points of week 2. l, m, n, o. The 12 mg/mL MNU case. m. On the OCT, unlike baseline (l), the ellipsoid zone, and outer nuclear layer (ONL) were indistinguishable at week 2. In addition, there was a decrease in INL thickness. n. The total retinal thickness of the 12 mg/mL MNU case at week 6 was thinner than at week 2. o. An OCT image at week 12 showed no significant change compared with the results at week 6. p, q. After week 2, the retina melted and induced retinal detachment in SD-OCT images of the 16 mg/mL MNU case. (SD-OCT = spectral domain optical coherence tomography; MNU = N-methyl-N-nitrosourea; GCL = ganglion cell layer; IPL = inner plexiform layer; INL = inner nuclear layer; OPL = outer plexiform layer; ONL = outer nuclear layer; EZ = ellipsoid zone; RPE = retinal pigment epithelium)

Supplementary Video 1. Intraoperative findings of two representative cases in 10mg/mL MNU group. First case is a representative case of severe retinal degeneration showing whole retina whitening during MNU tamponade. Second case is a representative case of moderate retinal degeneration. After 10 min of MNU solution tamponade, the color of the degenerated retina changed to white; this color change was not evident in the non-degenerated far periphery during vitrectomy

Supplementary Video 2. Surgical process for induction of retinal degeneration. The vitreous is removed using a vitreous cutter, while continually supplying balanced salt solution (BSS). After core vitrectomy, posterior vitreous detachment is induced gently to avoid an iatrogenic retinal break. And then, lensectomy is performed. Air-fluid exchange is then carried out. The vitreous cavity is fully tamponaded with different concentrations of MNU solution for 10 minutes, and MNU solution was removed by air-fluid exchange. Finally, the vitreous cavity was rinsed 3 times and filled with BSS to ensure complete removal of the MNU.
